# Effectiveness of near-infrared spectroscopy as a non-invasive tool to discriminate spectral profiles of *in vitro* cultured oocytes from goats

**DOI:** 10.1590/1984-3143-AR2020-0255

**Published:** 2021-12-10

**Authors:** Denilsa Pires Fernandes, Rafael Rossetto, Assis Rubens Montenegro, César Carneiro Linhares Fernandes, Pamela Angela Bravo, Maria Eugenia Moreno, Camila Muniz Cavalcanti, Guilherme Araújo Kubota, Davide Rondina

**Affiliations:** 1 Laboratório de Nutrição e Produção de Ruminantes, Faculdade de Veterinária, Universidade Estadual do Ceará, Fortaleza, CE, Brasil

**Keywords:** near infrared, spectroscopy, culture medium, oocyte, goat

## Abstract

Here, we aimed to discriminate between the spectral profiles of spent culture media after oocyte *in vitro* maturation (IVM) and culture (IVC) from goats of different ages subjected to repeated hormonal treatments. The profiles were discriminated using near infrared (NIR) spectroscopy combined with multivariate methods. A total of 19 goats (young = 10; old = 9) were subjected to serial hormonal stimulation (HS) with gonadotropins. Cumulus oophorus complexes (COCs) were collected using laparoscopic ovum pick-up (LOPU) and subjected to IVM and parthenogenetic activation. The initial embryos were subjected to IVC. Spent culture media were collected after oocyte IVM and on day 2 of IVC and analyzed using NIR spectroscopy. NIR spectral data were interpreted through chemometric methods, such as principle component analysis (PCA) and partial least square discriminant analysis (PLS-DA). The results of PCA analysis clearly showed a separation in the spectral profiles between the experimental groups (HS sessions; young and old animals) both after IVM and IVC. Overall, the main absorption bands were attributed to the C-H group second overtone, first overtone of O-H and N-H, and C-H combinations and may serve as molecular markers. On the other hand, the spectral data obtained using PLS-DA models provided a better classification of the groups. The results showed the possibility of discriminating young and old groups as well as the three HS sessions with high specificity, sensitivity, and accuracy using NIR spectra. Thus, the culture medium analysis using NIR spectroscopy combined with multivariate methods indicated the dissimilarities between the groups and provided an insight into the *in vitro* development of goat oocytes. This technique serves as an efficient, objective, rapid, and non-invasive method to discriminate spectral profiles.

## Introduction

Goats are important experimental models for *in vitro* embryo production (IVEP) and offer great advantages in the generation of transgenic animals as well as cloning and conservation studies of animals that are at risk of extinction (Koeman et al., 2003; Mariano et al., 2015). Subjecting young animals to IVEP may help reduce generation interval (Paramio, 2010) and increase the recovery rate of oocytes per ovary (Koeman et al., 2003). Despite its great potential, IVEP in goats may not be in compliance with other domestic species, leading to variations in success rates associated with embryos produced *in vivo* (Paramio, 2010; Freitas et al., 2017), especially among animals of different ages (Leoni et al., 2009, 2015).

The ability of an oocyte to successfully develop into an embryo is critical during the capacitation process, which precedes ovulation. During capacitation, the oocyte accumulates a set of information that can be used in the successive stages of development. The heterogeneity of oocytes collected for *in vitro* maturation (IVM) is still a limiting factor to the success of embryonic development in goats (Souza-Fabjan et al., 2014a). Some aspects or conditions such as oocyte source (Souza-Fabjan et al., 2014a), ovarian status and stimulation (Gibbons et al., 2007; Avelar et al, 2012), age (Souza-Fabjan et al., 2013), and season (Souza-Fabjan et al., 2014b) may contribute to oocyte heterogeneity and affect its developmental competence. The effects of age on IVEP in goats have been investigated and differences in some reproductive aspects between young and old goats were reported (Paramio, 2010; Baldassarre et al., 2007). Furthermore, the use of laparoscopic ovum pick-up (LOPU) technique and hormonal stimulation (HS) followed by IVEP has been effective in improving the poor reproductive performance of animals at very young age (Currin et al., 2017), especially in the animals that had their fertility affected with advanced age (Baldassarre et al., 2007).

Although morphological evaluation is very useful, it is highly subjective and may be unreliable for predicting the viability of these structures upon cultivation under *in vitro* conditions (Seli et al., 2008; Vergouw et al., 2008). Therefore, several studies have focused on the development of an improved method for gamete evaluation as the primary objective (Li et al., 2015). To address the main pitfalls in IVEP, a metabolomic profiling technology was proposed as an alternative to invasive techniques to enable simultaneous and high performance investigation of metabolic profiles based on the metabolites present in biological samples (McLennan et al., 2020; Leary and Sturmey, 2020). This technique evaluates the entire metabolic profile as a pattern and may be used for sample classification (Seli et al., 2010; Muñoz et al., 2014; Li et al., 2015).

Several technologies analyze spent culture media and suggest the presence of metabolic differences between oocytes or embryos, which mainly determine the reproductive potential (Ahlström et al., 2011; Muñoz et al., 2014; Rubessa et al., 2016). Optical spectroscopies as analytical technologies have been employed to investigate the complex metabolic profiles of culture media, including Raman, Fourier transform infrared (FT-NIR), and near infrared (NIR) spectroscopy (Botros et al., 2008, Ishigaki et al., 2014). Of these, the NIR spectroscopy has gained wide acceptance in different fields (Xiaobo et al., 2010), and is recognized as a primary and simple instrumentation that exposes the target sample to light of different wavelengths. It measures the characteristic absorption spectrum of the sample (Botros et al., 2008) and stands out owing to very fast and highly reproducible measurements, high sensibility, short and simple sample processing methods, easy maintenance and operation, and cost effectiveness (Botros et al., 2008). In this way, NIR technology provides a new and useful approach to oocyte and embryo evaluation using the spent medium culture in a non-invasive, non-toxic, and rapid analysis and processes small volumes compatible with the *in vitro* culture (IVC) system without preparation or prior separation.

NIR spectroscopy, in combination with chemometric tools, has demonstrated good results in the extraction of hidden information from simple spectra that may be difficult to interpret using computation of multivariate models (Rødgaard et al., 2015). These interpretations can detect minimal variations and accurately identify alterations in the metabolic spectra between oocytes (Nagy et al., 2009) from young and old animals, based on the differences in maturation rates and embryo production (Souza-Fabjan et al., 2013) as well as the changes in the quality of oocytes recovered by LOPU after repeated hormonal treatments (Gibbons et al, 2007; Avelar et al., 2012). Several studies have described the use of spectroscopy on spent culture media for human (Vergouw et al., 2008), mouse (Ishigaki et al., 2017), cattle (Muñoz et al., 2014; Li et al., 2018), fish (Ishigaki et al., 2016, 2017; Bik et al., 2020) and goat (Zhang et al., 2018) models. Thus, the use of NIR spectroscopy combined with multivariate analysis may accurately determine a metabolic pattern based on the spectral analysis of the oocyte maturation medium, that can be linked to oocyte subsequent metabolism and viability and improve *in vitro* culture system for assisted reproduction. In this way, predicting the potential of implantation and consequent pregnancy may be possible (Nagy et al., 2009).

Herein, we propose the use of NIR spectroscopy in combination with multivariate analysis as a technique for the non-invasive discrimination between the spectral profiles of spent culture media after IVM and IVC of oocytes obtained from goats of different ages subjected to repeated hormonal treatments.

## Materials and methods

### Animals

A total of 19 mixed-breed goats were selected as oocyte donors, 10 aged between 1.3 ± 0.2 years and weighing 26.5 ± 2.0 kg, and nine non-lactating goats, aged between 7.0 ± 1.0 years and weighing 42.8 ± 2.1 kg. Both groups exhibited homogenous body condition scores (2.8 ± 0.1) on a scale of 1 to 5 adapted from Morand–Fehr and Gall (1981). The goats were allocated in the Experimental Farm of School Veterinary Medicine, State University of Ceará (UECE), located in Guaiúba, CE, in the equatorial zone (4º2’23”S and 38º38’14”W) and subjected to 30–45 days of adaptation to environmental and food conditions with balanced diet as per their nutritional requirements.

### Hormonal treatment

Estrus synchronization and ovarian stimulation were performed according to protocols described by Gibbons et al. (2007).

#### Estrous synchronization

Briefly, the ovarian status was synchronized in all goats with an intramuscular administration of 1 ml (0.075 mg) of a prostaglandin F2α analogue (PGF2α; Prolise^®^; ARSA S.R.L., Buenos Aires, Argentina), which was followed by the insertion of an intravaginal progesterone insert (CIDR^®^, InterAg^®^, Hamilton, New Zealand) after 48 h. The intravaginal insert was removed after the last oocyte recovery.

#### Hormonal stimulation

Follicular development was stimulated by the simultaneous intramuscular administration of a single dose of 60 mg follicle-stimulating hormone (FSH; Folltropin^®^; Vetrepharm, London, ON, Canada) along with a single dose of 300 UI equine chorionic gonadotropin (eCG; Novormon 5000^®^; Syntex, Argentina). The first FSH/eCG dose was administered 48 h after the dispositive insertion and repeated every 4 days to complete three laparoscopic LOPU sessions.

### LOPU and oocyte evaluation

The oocyte donors were divided into four groups (4-5 animals per group) and subjected to three sessions of HS and subsequent LOPU with a 4-day time interval between the sessions (Gibbons et al., 2007). LOPU was performed 24 h after each FSH/eCG treatment and 36 h of fasting. The follicular fluid from each animal was separately aspirated into collection tubes and warmed to 38°C in Dulbecco’s phosphate-buffered saline (D-PBS; Nutricell^®^, Campinas, SP, Brazil), which was supplemented with 5% fetal bovine serum (FBS; Sigma Chemical Co., St Louis, MO, USA), antibiotics (100 IU/ml penicillin plus 0.1 mg/ml streptomycin; Sigma Chemical Co., St Louis, MO, USA), and 0.05 mg/ml heparin (Liquemine®, Campinas-SP, Brazil). The follicular fluid sediment was used to recover cumulus oophorus complexes (COCs) that were classified according to the method described by Baldassarre et al. (2003) with minor modifications. Briefly, the assessment of the quality of COCs was based on visual criteria observed using a stereomicroscope (SMZ-645; Nikon^®^, Tokyo, Japan) and classified into four different grades as follows: Grade 1 (GI; oocyte with multilayered compact cumulus cells and evenly homogeneous cytoplasm); Grade 2 (GII; oocyte with 2-3 layers of cumulus cells and evenly homogeneous cytoplasm); Grade 3 (GIII; oocyte with 1 layer of cumulus cells, partially or totally denuded with homogeneous cytoplasm); and degenerated (DEG; denuded oocyte with heterogeneous cytoplasm or cumulus expansion).

### IVM, parthenogenetic activation, and IVC

Selected COCs, segregated as viable by grade (G-I to G-III) according to morphological quality and experimental groups, were subjected to IVM, parthenogenetic activation and IVC procedures according to Fernandes et al. (2014), with minor modifications. Briefly, COCs were subjected to IVM at 38.5°C and 5% CO_2_ in petri dishes (Corning^®^, USA) under mineral oil-containing drops of 100 µL of maturation medium. The medium comprised TCM 199^®^ medium supplemented with 0.022 µg/ml sodium pyruvate, 10,000 IU penicillin, 10,000 µg/ml streptomycin sulfate, 10% FBS, 10 ng/ml epidermal growth factor (EGF), 5 µg/ml FSHp (Folltropin^®^; Bioniche, Belleville, Ontario, Canada), 10 µg/ml luteinizing hormone (LH; Lutropin^®^; Bioniche), 1 µg/ml 17β-estradiol, and 100 µM cysteamine. After 24 h of IVM, oocytes were mechanically denuded with successive pipetting and observed under a stereomicroscope to determine the maturation rate through the careful visualization of the presence of the first polar body (metaphase II-MII). The *in vitro*-matured oocytes were parthenogenetically activated after exposure to 5 mM ionomycin for 5 min, followed by incubation in 2 mM 6-dimethylaminopurine (6-DMAP) in G1 (Vitrolife^®^) medium for 4 h. After activation, the presumed zygotes were cultured in G1 medium in an incubator at 38.5°C and 5% CO_2_ in a humidified atmosphere for 72 h. The embryos were subsequently incubated in 100-µL droplets of TCM199-HEPES with 10 M Hoechst 33342 (Sigma^®^, Deisenhofen, Germany) at 38.5°C for 30 min and individually examined under a fluorescence microscope (Nikon^®^, Eclipse 80i, Tokyo, Japan) to visualize cellular DNA and determine the cleavage rate.

### Sample collection and NIR spectroscopy analysis

After IVM of oocytes and IVC of presumed zygotes, 25 µL aliquots of the spent culture medium from each animal and blank controls (medium cultured without oocytes or embryos) were individually stored in correctly identified cryovials at −80°C for further analysis using NIR spectroscopy equipment (Perten DA 7200^©^). The medium was analyzed as microdrops covered with mineral oil in a petri dish. Before analysis, samples were thawed at room temperature (25°C ± 1°C) and evaluated in triplicate for spectral identification using NIRS. These spectra were recorded in the wavelength range between 950 and 1,650 nm (10526-6061 cm^−1^) at room temperature and collected at 5 nm intervals. The equipment was set to measure the raw transmittance and a signal average of 30 measurements with an integration time of 100 ms for each measurement. The analysis time was approximately 5 min per sample. The background spectrum (i.e., blank controls) was also obtained using the same acquisition time and power.

### Statistical analysis

The data were transformed into Excel 2010 and imported into the R programming environment (R Core Team, 2019). The spectral data were subjected to transformation with the second derivative to adjust baseline errors. Principal component analysis (PCA) was used to reduce the dimensionality of the data. The first three main components (uncorrelated independent variables) were used for sample clustering. Thus, it was possible to visualize all the spectra through the scores in a multidimensional space. We used the partial least square-discriminant analysis (PLS-DA) to predict the different classes. The PLS-DA algorithm maximizes the covariance between the spectra and the sample groups (age and HS sessions) through the successive selection of orthogonal latent variables (LVs). Moreover, variable importance in the projection (VIP) values obtained from supervised statistical PLS-DA was used to quantify the contribution of each wavelength in a PLS-DA model. According to the criteria of a VIP value > 1.0 in the PLS-DA model (Gosselin et al., 2010).

Cross-validation was performed 100 times in an iterative process to estimate the classification error rate to evaluate the performance of model by function perf, into the mixOmics package (Rohart et al., 2017). The samples were randomly divided (k-fold = 10) into 10 groups of equal sizes (MIV = 11 and CIV = 12), where nine were used to the training set and one to the test set. Moreover, to determine the predictive capacity of the model, the receiver operating characteristic (ROC) curve was used to evaluate the cutoff value by combining the highest specificity and sensitivity, which allowed classification of age groups and serial hormonal stimulation (HS) groups through the area under the curve (AUC).

### Ethics approval

All procedures used in this study were conducted as per the Brazilian Directive on the Care and Use of Animals in Teaching or Scientific Research Activities (DBCA (Chapter VI, item 6.3.10). The study was approved by the Ethics Committee for the use of animals of the State University of Ceará under registration order number 6497021/2017.

## Results

**Figure 1:**
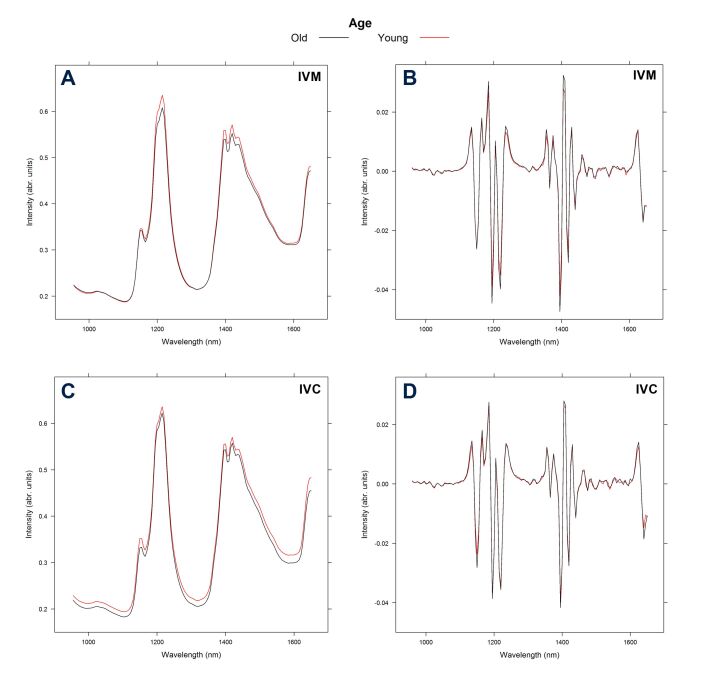
Raw (A) and second derivative (B) NIR spectra of spent culture medium samples after IVM. Raw (C) and second derivative (D) NIR spectra of spent culture medium after IVC of oocytes from old (black line) and young (red line) goats.

**Figure 2:**
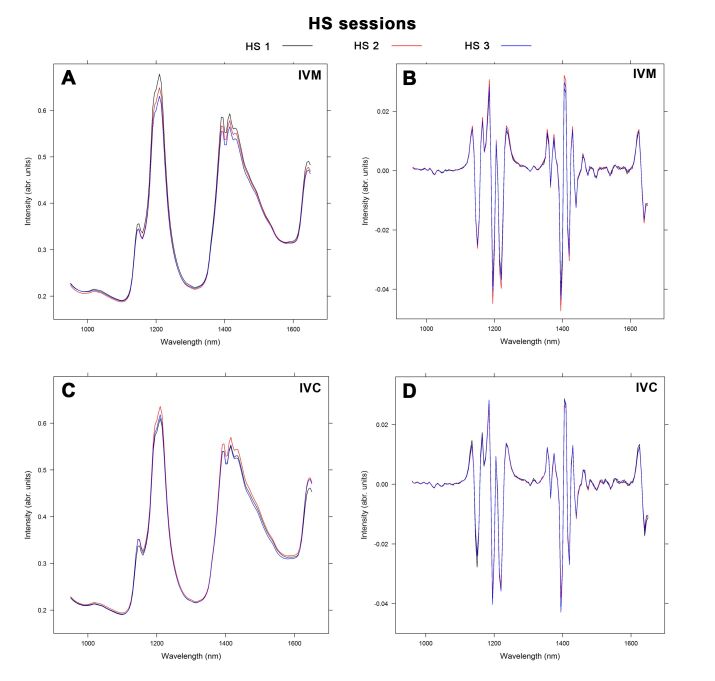
Raw (A) and second derivative (B) NIR spectra of spent culture medium samples after IVM. Raw (C) and second derivative (D) NIR spectra of spent culture medium after IVC of oocytes from the first (black line), second (red line), and third (blue line) hormonal stimulation (HS) session.

Figure 1 shows the raw and second derivative spectral profiles after IVM (Figure 11B, respectively) and IVC (Figure 11D, respectively) obtained using NIR spectroscopy. The results were obtained from the analysis of culture spent media of young and old animals. In Figure 2, the raw and second derivative NIR spectra are shown after IVM (Figure 22B, respectively) and IVC (Figure 22D, respectively) in response to the three HS sessions. The spectra obtained showed six main absorption bands, 1,150, 1,220, 1,400, 1,425, 1,445 and 1,650 nm, attributed to the vibration of C-H group second overtone, first O-H and N-H overtone, and C-H combinations. Evaluation of the second derivative-treated spectra revealed more sharp peaks and valleys at 1,130-1,225, 1,610-1,625, and 1,355-1,460 nm range. The spectral profile of young and old animals as well as the samples from different HS sessions showed different absorption patterns in different regions.

**Figure 3:**
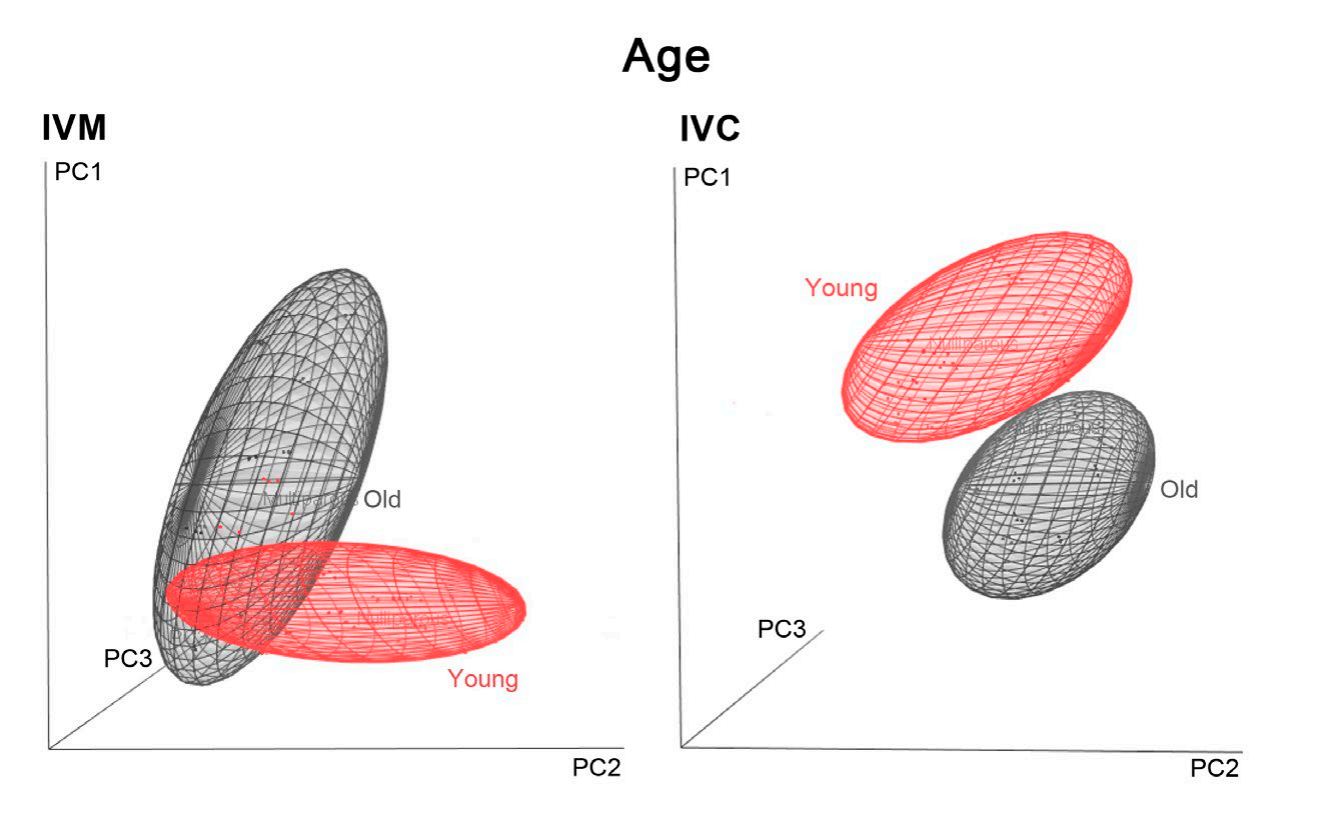
Score plot of PCA model with three principal components of spent culture medium spectra of oocytes from the young (red) and old groups (black) after IVM (left) and IVC (right).

**Figure 4:**
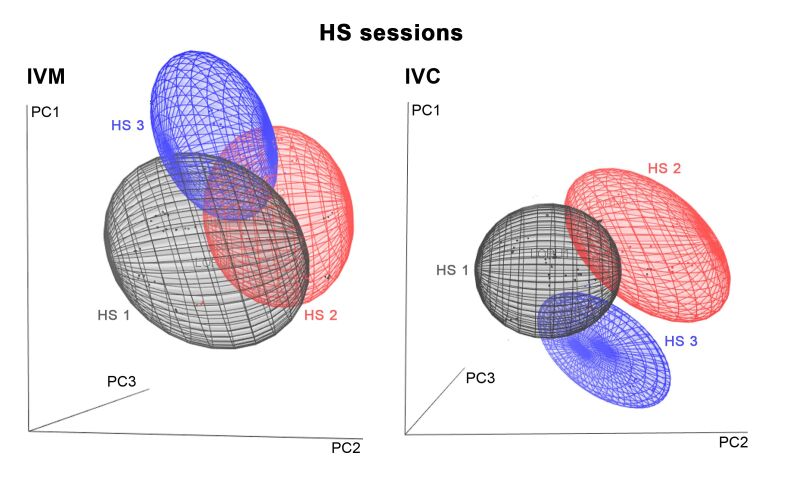
Score plot of PCA model with three principal components of spent culture medium spectra of oocytes obtained from the first (black line), second (red line), and third (blue line) hormonal stimulation (HS) session after IVM (left) and IVC (right).

Figure 3 presents the three-dimensional score plot of the first three PCs of the different age groups (young and old) for IVM (Figure 3A) and IVC (Figure 3B) phases obtained through PCA. Figure 4 shows the score plot of the three HS sessions for IVM (Figure 4A) and IVC (Figure 4B). Analysis of multivariate was capable to segregate the groups in age and HS effects. Multivariate retained for the first, second and third principal components respectively 17%, 12%, and 11% of the total variance in IVM model and for IVC 18%, 13%, and 12% of variance, accounting for respectively 40% and 43% of the cumulative variance together.

**Figure 5:**
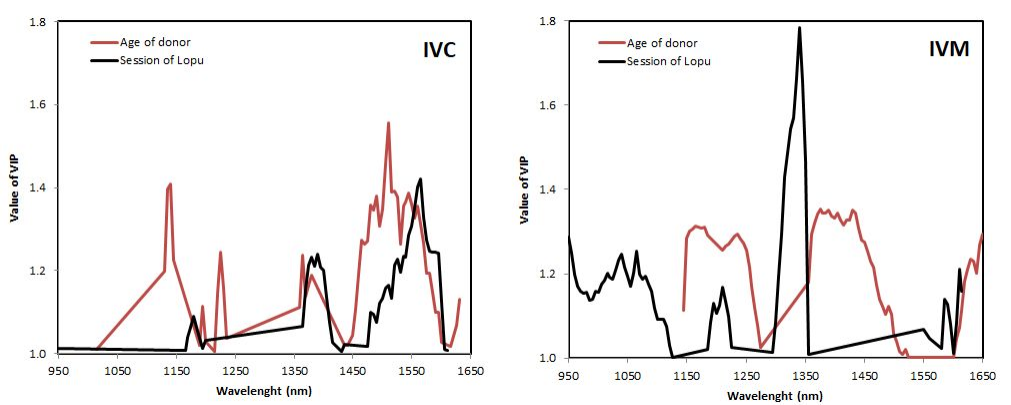
Distribution curves of variable importance in the projection (VIP) values > 1.0 obtained from the PLS-DA multivariate model of spent culture medium spectra of oocytes obtained from goats with different ages (red) and hormonal stimulation sessions (black) after IVM (A) and IVC (B).

In Figure 5, were illustrated the values of variable importance in the projection (VIP) for IVM (Figure 5A) and IVC (Figure 5B). The IVM VIPs shows a distinct pattern distribution for age and hormonal stimulation sessions. In HS curve there was a highest value (1.78) at 1,340 nm wavelengths and VIPs range distributed from 950 nm to 1,615nm. On the contrary, the age curve shows VIPs from 1,145 nm to 1,650 nm, with two peaks located at 1,200 nm and 1,400 nm but both always showing VIP below 1.4.

The distribution curves of the VIPs in the IVC showed values below 1.6 and with similar patterns. Due to areas of larger peaks, they can be identified between 1,360 nm and 1,400 nm and successively between 1,460 nm and 1,600 nm. The age of the animals also registered a smaller peak between 1,135 nm and 1,145 nm.

**Figure 6:**
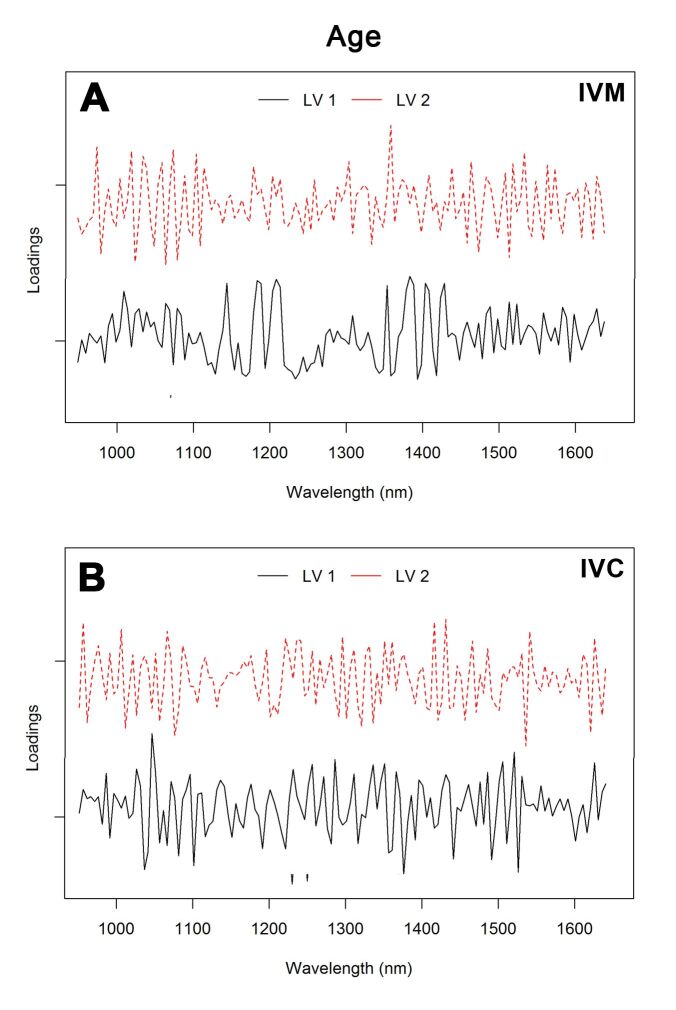
Loading plot of the first two latent variables (LVs) for the PLS-DA model of spent culture medium spectra of oocytes obtained from the young and old groups after IVM (A) and IVC (B).

Considering the groups of young and old animals, the error rates of PLS-DA with five LVs were 0.21 and 0.04 for IVM and IVC, respectively. The AUC for IVM was 86%, while that for IVC was 100%. The loading vectors of the first two LVs for the PLS-DA model considering the age group are provided in Figure 6. The main regions that contributed to this result included 1,075, 1,085, 1,370, 1,395 and 1,525 nm for IVM phase and 1,045, 1,055, 1,110, 1,385 and 1,535 nm for IVC.

**Figure 7:**
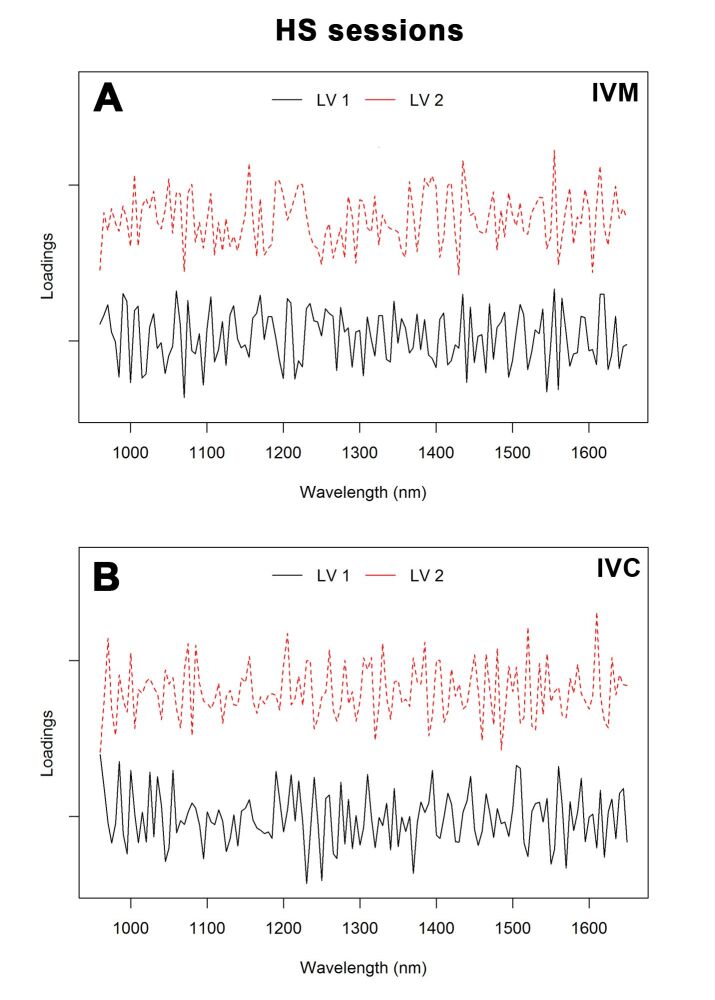
Loading plot of the first two latent variables (LVs) for the PLS-DA model of culture medium spectra of oocytes obtained from hormonal stimulation (HS) sessions after IVM (A) and IVC (B).

For HS sessions, error rates were 0.39 and 0.19 for IVM and IVC, respectively. The AUC was 94% (HS 1 versus others), 96% (HS 2 versus others) and 82% (HS 3 versus others) for IVM and 96% (HS 1 versus others), 98% (HS 2 versus others) and 100% (HS 3 versus others) for IVC. The loading plot (Figure 7) showed the main bands, which allowed the discrimination between groups subjected to different HS sessions. The main regions that contributed to the separation of HS sessions were 1,070, 1,305, 1,325, 1,555 and 1,560 nm for IVM and 960, 985, 1,230, 1,520 and 1,610 nm for IVC.

## Discussion

One of the known advantages of NIR technology is the ability to construct characteristic spectra, which serve as the “fingerprint” of the sample (Currà et al., 2019). Here, we used NIR analysis for overall spectral profile comparisons, with an aim to distinguish between young and old groups and first, second and third HS session without further metabolic investigation. We found that the technique in conjunction with multivariate analysis was capable of separating the experimental groups. During *in vitro* development, oocytes and embryos may secrete/absorb certain levels of substances into/from the medium culture, such valine, lysine, glutamine, glycine, phenylalanine, phytosphingosine, lactate, glucose, pyruvate and phosphate (Zhang et al., 2018; Wiweko et al., 2020). NIR spectroscopy is not suitable for structure elucidation; therefore, band attribution is recommended for spectral interpretation and for a better understanding of the results. This includes the use of band characteristics such as intensity and wavelength position for correlating chemical knowledge about the sample in an attempt to identify the corresponding compound (Pasquini, 2003).

In our study, young and old animals were subjected to sequential HS for the evaluation of oocyte and embryo quality. The effectiveness of ovarian stimulatory protocols on maturation and cleavage rates is well known (Baldassarre and Karatzas, 2004; Paramio, 2010; Baldassarre et al., 2018), and studies have evaluated the effects of repeated hormonal treatments on the morphological quality of recovered oocytes (Gibbons et al., 2007).

Overall, the NIR spectrum provides information about the spectral signatures of biological samples (Chen et al., 2015). The wavelength range evaluated in this work comprises the region between 950 and 1,650 nm, wherein four functional groups (C-H, N-H, O-H, and C-C) can be monitored. The spectra were different in some regions, reflective of the vibration, overtone, and combinations of functional groups such as O-H, N-H, and C-H. These overtones are anharmonic, leading to very broad, complex spectra that are not directly interpretable (Nagy et al., 2009). In addition, molecules have different vibration patterns, leading to the requirement of highly complex and sensitive equipment for the identification of a large number of molecules. Previous studies have reported differences in the absorbance patterns in NIR spectra by evaluating culture media obtained from oocytes at different maturation stages (Nagy et al, 2009) and single embryo culture for viability assessment (Seli et al., 2007; Li et al, 2015).

In the present study, the NIR spectra were pretreated with the second derivative to extract important information and minimize variability (Chen et al., 2015). The second derivative technique is a common filter used in practice to focus on the variation in the spectra associated with the chemical composition of the sample (Kirdar et al., 2010). Therefore, this technique was considered as a suitable choice for the pre-treatment of NIR spectra for subsequent multivariate calibration. The second derivative spectra in the range of the wavelength 1,130–1,225, 1,355–1,460, and 1,610–1,625 nm demonstrated changes in the peak bands. According to Chen et al. (2015), the absorption range between 1,111 and 1,250 nm is associated with C-H second overtones, while the range between 1,333 and 1,666 nm is attributed to the first overtone of O-H and N-H, and C-H combinations. As observed in Figure 11D, several overlapping vibrational bands were detected in the two groups of spectra (young and old animals) at both times of evaluation (after IVM and IVC). Furthermore, several sharp peaks and valleys were detected, which were not observed with raw spectra. This observation is indicative of the high information content in the NIR spectra, which may explain the variation in the absorption bands that may not be easy to distinguish or attribute to certain functional groups. According to Huang et al. (2010), the derivative technique can prove difficult for spectral interpretation and may possibly lead to more noisy spectra. It is worth noting that the peak wavelengths were similar between age groups, and that some absorbance peaks in culture medium spectra after IVC were more pronounced than those after IVM (Fig. 1).

The use of multivariate statistical techniques such as PCA and PLS-DA enabled the extraction of useful information from NIR spectra. PCA was conducted to review the data from spectral profiles of the investigated groups and to examine the possible clustering in samples. Our results demonstrated a good separation between different age groups in the culture media after both IVM and IVC. Differentiated metabolism can be influenced by significant deficiencies in the number and organization of organelles in young animal oocytes, wherein reduction in the amount of endoplasmic reticulum has been observed (Damiani et al., 1996). According to Baldassarre et al. (2018), multiple applications of gonadotrophins for follicular development, especially in young animals, improved the cytoplasmic conditions of competent oocytes and consequently the cleavage rates. This result reveals the complexity of the physiological and biochemical mechanisms involved in the *in vitro* development of goat oocytes. Nevertheless, a more careful assessment using NIR analysis would reveal the distinct patterns of secretions in the experimental groups based on spectral evaluations of spent culture media and uncover previously unseen differences (Kirkegaard et al., 2014).

The first three PCs showed about 43% of total variation in the NIR spectra. These results needed to be further analyzed using PLS-DA to maximize the separation between the groups and provide a better understanding for classification (i.e., dimensionality reduction) and predicting model construction (i.e., discriminant analysis) (Lee et al., 2018). We applied the classification error rate, ROC curve and AUC as figures of merit and constructed an ROC curve to help select a threshold (cutoff value), which was used to obtain high sensitivity, specificity, and overall accuracy. ROC-AUC is considered a powerful tool for the detection of small differences between groups and accurately finds the main variables responsible for discrimination between two classes (Szymanska et al., 2012). As a categorical model, these tools are valid for describing tlhe predictive ability of the PLS-DA model (Gromski et al., 2015). In our findings, the PLS-DA model showed consistent AUC values, which were higher than 0.8. For the IVC phase, excellent AUC value was obtained (1.0: perfect discrimination). The figures of merit indicated that the classification model established herein was characterized with high accuracy and acceptable values of errors. Therefore, it can be stated that PLS-DA was an efficient and reliable model with excellent discriminatory power for the comparison of the culture media of young and old animal oocytes after IVC. Moreover, the loading plot in Figure 6A shows that LV1 focused on the second overtone of C-H in –C-H_2_, O-H, and N-H first overtones, and C-H combination regions, while LV2 focused on the second overtone C-H stretching and H_2_O bands.

Oocytes from young animals subjected to hormonal treatments had greater developmental capacity and may potentially combine the effect of older age and multiple gonadotropin stimulations (Baldassarre et al., 2018). Our results showed six main absorption peaks in NIR spectra, 1,150, 1,220, 1,400, 1,425, 1,445, and 1,645 nm, for the three groups, that may be attributed to the vibration of the C-H group second overtone, first O-H and N-H overtone, and C-H combinations. PCA showed that the three HS sessions were well clustered (Figure 4), and PC1 resolved most of the variance between them. Furthermore, the results of the PLS-DA procedure to discriminate the experimental groups achieved the highest sensitivity and specificity, best accuracy values, and low error rates. These efficient classification results suggest that the procedure was reliable, and that the classification models were robust. Our loading plots support these results (Figure 6), revealing the dominating or influencing variables. As shown in Figure 6B, LV1 focused on the water bands, C-H second overtone, and O-H first overtone. The region between 1,070, 1,305, and 1,325, and 1,555 and 1,560 nm for IVM and 960 and 985, and 1,230, 1,520, and 1,610 nm for IVC contributed to the differentiation of various classes. It is worth noting that some absorption bands were difficult to assign, mostly owing to the absence of previous identification or band overlaps.

## Conclusion

We conclude that NIR spectroscopy is a rapid and non-invasive technique for the analysis of culture media used for *in vitro* goat oocyte maturation and development. In combination with multivariate methods, NIR spectroscopy serves as a useful tool for the classification and separation of mature and cleaved oocytes from young and old goats as well as from those subjected to repeated HS at each session. PCA enabled the separation of the experimental groups, while the PLS-DA model enabled the prediction of sample classes with high accuracy. The results of this work show that the NIR spectra may provide relevant information on IVM and IVC and contribute to the high efficiency of the technique.

## References

[B001] Ahlström A, Wikland M, Rogberg L, Barnett JS, Tucker M, Hardarson T (2011). Cross-validation and predictive value of near-infrared spectroscopy algorithms for day-5 blastocyst transfer. Reprod Biomed Online.

[B002] Avelar SRV, Moura RR, Sousa FC, Pereira AF, Almeida KC, Melo CHS (2012). Oocyte production and *in vitro* maturation in Canindé goats following hormonal ovarian stimulation. Anim Reprod.

[B003] Baldassarre H, Currin L, Michalovic L, Bellefleur AM, Gutierrez K, Mondadori RG, Glanzner WG, Schuermann Y, Bohrer RC, Dicks N, Lopez R, Grand FX, Vigneault C, Blondin P, Gourdon J, Bordignon V (2018). Interval of gonadotropin administration for *in vitro* embryo production from oocytes collected from Holstein calves between 2 and 6 months of age by repeated laparoscopy. Theriogenology.

[B004] Baldassarre H, Karatzas CN (2004). Advanced assisted reproduction Technologies (ART) in goats. Anim Reprod Sci.

[B005] Baldassarre H, Rao KM, Neveu N, Brochu E, Begin I, Behboodi E, Hockley DK (2007). Laparoscopic ovum pick-up followed by *in vitro* embryo production for the reproductive rescue of aged goats of high genetic value. Reprod Fertil Dev.

[B006] Baldassarre H, Wang B, Kafidi N, Gauthier M, Neveu N, Lapointe J, Sneek L, Leduc M, Duguay F, Zhou JF, Lazaris A, Karatzas CN (2003). Production of transgenic goats by pronuclear microinjection of *in vitro* produced zygotes derived from oocytes recovered by laparoscopy. Theriogenology.

[B007] Bik E, Ishigaki M, Blat A, Jasztal A, Ozaki Y, Malek K, Baranska M (2020). Lipid droplet composition varies based on medaka fish eggs development as revealed by NIR-, MIR-, and Raman Imaging. Molecules.

[B008] Botros L, Sakkas D, Seli E (2008). Metabolomics and its application for non-invasive embryo assessment in IVF. Mol Hum Reprod.

[B009] Chen H, Lin Z, Mo L, Wu T, Tan C (2015). Near-infrared spectroscopy as a diagnostic tool for distinguishing between normal and malignant colorectal tissues. BioMed Res Int.

[B010] Currà A, Gasbarrone R, Cardillo A, Trompetto C, Fattapposta F, Pierelli F, Missori P, Bonifazi G, Serranti S (2019). Near-infrared spectroscopy as a tool for *in vivo* analysis of human muscles. Sci Rep.

[B011] Currin L, Michalovic L, Bellefleur AM, Gutierrez K, Glanzner W, Schuermann Y, Bohrer RC, Dicks N, da Rosa PR, De Cesaro MP, Lopez R, Grand FX, Vigneault C, Blondin P, Gourdon J, Baldassarre H, Bordignon V (2017). The effect of age and length of gonadotropin stimulation on the *in vitro* embryo development of Holstein calf oocytes. Theriogenology.

[B012] Damiani P, Fissore RA, Cibelli JB, Long CR, Balise JJ, Robl JM, Duby RT (1996). Evaluation of developmental competence, nuclear and ooplasmic maturation of calf oocytes. Mol Reprod Dev.

[B013] Fernandes CCL, Feltrin C, Martins LT, Gaudêncio S, Aguiar LH, Silva AM, Oliveira CH, Silva LM, Silva CM, Bertolini M, Rondina D (2014). Goat oocyte quality and competence to undergo IVM and embryo development after parthenogenetic activation from goats fed with different levels of cashew nut bran as source of dietary lipids. Theriogenology.

[B014] Freitas VJF, Souza-Fabjan JMG, Mermillod P, Melo LM, Teixeira DIA (2017). Estado da arte e perspectivas da produção *in vitro* de embriões em caprinos. Rev Bras Reprod Anim.

[B015] Gibbons A, Pereyra Bonnet F, Cueto MI, Catala M, Salamone DF, Gonzalez-Bulnes A (2007). Procedure for maximizing oocyte harvest for *In Vitro* embryo production in small ruminants. Reprod Domest Anim.

[B016] Gosselin R, Rodrigue D, Duchesne C (2010). A bootstrap-VIP approach for selecting wavelength intervals in spectral imaging application. Chemom Intell Lab Syst.

[B017] Gromski PS, Muhamadali H, Ellis DI, Xu Y, Correa E, Turner ML, Goodacre R (2015). A tutorial review: metabolomics and partial least squares-discriminant analysis–a marriage of convenience or a shotgun wedding. Anal Chim Acta.

[B018] Huang J, Romero-Torres S, Moshgbar M (2010). Raman: practical considerations in data pre-treatment for Nir and Raman spectroscopy. Am Pharm Rev..

[B019] Ishigaki M, Hashimoto K, Sato H, Ozaki Y (2017). Non-destructive monitoring of mouse embryo development and its qualitative evaluation at the molecular level using Raman spectroscopy. Sci Rep.

[B020] Ishigaki M, Kawasaki S, Ishikawa D, Ozaki Y (2016). Near-Infrared Spectroscopy and Imaging Studies of Fertilized Fish Eggs: In Vivo Monitoring of Egg Growth at the Molecular Level. Sci Rep.

[B021] Ishigaki M, Taketani A, Sato H (2014). Discrimination of fish egg quality and viability by Raman spectroscopy. Anal Methods.

[B022] Kirdar AO, Chen G, Weidner J, Rathore AS (2010). Application of near‐infrared (NIR) spectroscopy for screening of raw materials used in the cell culture medium for the production of a recombinant therapeutic protein. Biotechnol Prog.

[B023] Kirkegaard K, Svane ASP, Nielsen JS, Hindkjær JJ, Nielsen NC, Ingerslev HJ (2014). Nuclear magnetic resonance metabolomic profiling of Day 3 and 5 embryo culture medium does not predict pregnancy outcome in good prognosis patients: a prospective cohort study on single transferred embryos. Hum Reprod.

[B024] Koeman J, Keefer CL, Baldassarre H, Downey BR (2003). Developmental competence of prepubertal and adult goat oocytes cultured in semi-defined media following laparoscopic recovery. Theriogenology.

[B025] Leary C, Sturmey RG (2020). Metabolic profile of in vitro derived human embryos is not affected by the mode of fertilization. Mol Hum Reprod.

[B026] Lee LC, Liong CY, Jemain AA (2018). Partial least squares-discriminant analysis (PLS-DA) for classification of high-dimensional (HD) data: a review of contemporary practice strategies and knowledge gaps. Analyst.

[B027] Leoni GG, Palmerini MG, Satta V, Succu S, Pasciu V, Zinellu A, Carru C, Macchiarelli G, Nottola SA, Naitana S, Berlinguer F (2015). Differences in the kinetic of the first meiotic division and in active mitochondrial distribution between prepubertal and adult oocytes mirror differences in their developmental competence in a sheep model. PLoS One.

[B028] Leoni GG, Succu S, Satta V, Paolo M, Bogliolo L, Bebbere D, Spezzigu A, Madeddu M, Berlinguer F, Ledda S, Naitana S (2009). *In vitro* production and cryotolerance of prepubertal and adult goat blastocysts obtained from oocytes collected by laparoscopic oocyte-pick-up (LOPU) after FSH treatment. Reprod Fertil Dev.

[B029] Li X, Xu Y, Fu J, Zhang WB, Liu SY, Sun XX (2015). Non-invasive metabolomic profiling of embryo culture media and morphology grading to predict implantation outcome in frozen-thawed embryo transfer cycles. J Assist Reprod Genet.

[B030] Li XX, Cao PH, Han WX, Xu YK, Wu H, Yu XL, Chen JY, Zhang F, Li YH (2018). Non-invasive metabolomic profiling of culture media of ICSI-and IVF-derived early developmental cattle embryos via Raman spectroscopy. Anim Reprod Sci.

[B031] Mariano RSG, Uscategui RAR, Nociti RP, da Camara Barros FFP, Feliciano MAR, Rodriguez MGK, Taira AR, Teixeira PPM, Vicente WRR (2015). Aspiração folicular em ruminantes–revisão de literatura. Investigação..

[B032] McLennan HJ, Saini A, Dunning KR, Thompson JG (2020). Oocyte and embryo evaluation by AI and multi-spectral auto-fluorescence imaging: livestock embryology needs to catch-up to clinical practice. Theriogenology.

[B033] Morand-Fehr P, Gall C (1981). Nutrition and feeding of goats: application to temperate climatic condition; Goat production..

[B034] Muñoz M, Uyar A, Correia E, Díez C, Fernandez-Gonzalez A, Caamaño JN, Trigal B, Carrocera S, Seli E, Gomez E (2014). Non-invasive assessment of embryonic sex in cattle by metabolic fingerprinting of *in vitro* culture medium. Metabolomics.

[B035] Nagy ZP, Jones-Colon S, Roos P, Botros L, Greco E, Dasig J, Behr B (2009). Metabolomic assessment of oocyte viability. Reprod Biomed Online.

[B036] Paramio MT (2010). *In vivo* and *in vitro* embryo production in goats. Small Rumin Res.

[B037] Pasquini C (2003). Near infrared spectroscopy: fundamentals, practical aspects and analytical applications. J Braz Chem Soc.

[B038] R Core Team (2019). R: a language and environment for statistical computing.

[B039] Rødgaard T, Heegaard PMH, Callesen H (2015). Non-invasive assessment of *in vitro* embryo quality to improve transfer success. Reprod Biomed Online.

[B040] Rohart F, Gautier B, Singh A, Lê Cao K-A (2017). MixOmics: an R package for ‘omics feature selection and multiple data integration. PLOS Comput Biol.

[B041] Rubessa M, Ambrosi A, Denmark SE, Wheeler MB (2016). Non-invasive analysis of gamete metabolites during *in vitro* embryo production using nuclear magnetic resonance. International Journal of New Technology and Research..

[B042] Seli E, Botros L, Sakkas D, Burns DH (2008). Non invasive metabolomic profiling of embryo culture media using proton nuclear magnetic resonance correlates with reproductive potential of embryos in women undergoing *in vitro* fertilization. Fertil Steril.

[B043] Seli E, Robert C, Sirard M (2010). OMICS in assisted reproduction: possibilities and pitfalls. Mol Hum Reprod.

[B044] Seli E, Sakkas D, Scott R, Kwok SC, Rosendahl SM, Burns DH (2007). Noninvasive metabolomic profiling of embryo culture media using Raman and near-infrared spectroscopy correlates with reproductive potential of embryos in women undergoing *in vitro* fertilization. Fertil Steril.

[B045] Souza-Fabjan JMG, Locatelli Y, Duffard N, Corbin E, Touzé JL, Perreau C, Beckers JF, Freitas VJF, Mermillod P (2014). *In vitro* embryo production in goats: slaughterhouse and laparoscopic ovum pick up-derived oocytes have different kinetics and requirements regarding maturation media. Theriogenology.

[B046] Souza-Fabjan JMG, Panneau B, Duffard N, Locatell Y, Figueiredo JR, Freitas VJF, Mermillod P (2014). *In vitro* production of small ruminant embryos: late improvements and further research. Theriogenology.

[B047] Souza-Fabjan JMG, Pereira AF, Melo CH, Sanchez DJ, Oba E, Mermillod P, Melo LM, Teixeira DIA, Freitas VJ (2013). Assessment of the reproductive parameters, laparoscopic oocyte recovery and the first embryos produced *in vitro* from endangered Canindé goats (*Capra hircus*). Reprod Biol.

[B048] Szymańska E, Saccenti E, Smilde AK, Westerhuis JA (2012). Double-check: validation of diagnostic statistics for PLS-DA models in metabolomics studies. Metabolomics.

[B049] Vergouw CG, Botros LL, Roos P, Lens JW, Schats R, Hompes PGA, Burns DH, Lambalk CB (2008). Metabolomic profiling by near-infrared spectroscopy as a tool to assess embryo viability: a novel, non-invasive method for embryo selection. Hum Reprod.

[B050] Wiweko B, Zakia Z, Tedjo A, Widyahening IS, Pratama G, Hestiantoro A, Natadisastra M, Sumapraja K, Harzif AK, Zakirah SC (2020). Prediction of good quality blastocyst formation by metabolomic profile of spent embryo culture media using FTIR Spectroscopy in Women undergoing IVF Cycle: a Cohort prospective study. Res Sq.

[B051] Xiaobo Z, Jiewen Z, Povey MJ, Holmes M, Hanpin M (2010). Variables selection methods in near-infrared spectroscop. Anal Chim Acta.

[B052] Zhang YL, Zhang GM, Jia RX, Wan YJ, Yang H, Sun LW, Han L, Wang F (2018). Non‐invasive assessment of culture media from goat cloned embryos associated with subjective morphology by gas chromatography–mass spectroscopy‐based metabolomic analysis. Anim Sci J.

